# HIV-1 Infection-Induced Suppression of the Let-7i/IL-2 Axis Contributes to CD4^+^ T Cell Death

**DOI:** 10.1038/srep25341

**Published:** 2016-05-05

**Authors:** Yijun Zhang, Yue Yin, Shaoying Zhang, Haihua Luo, Hui Zhang

**Affiliations:** 1 Institute of Human Virology, Zhongshan School of Medicine, Sun Yat-sen University, Guangzhou, Guangdong, 510080, China; 2 Key Laboratory of Tropical Disease Control of Ministry of Education, Zhongshan School of Medicine, Sun Yat-sen University, Guangzhou, Guangdong, 510080, China

## Abstract

The mechanisms underlying HIV-1-mediated CD4^+^ T cell depletion are highly complicated. Interleukin-2 (IL-2) is a key cytokine that maintains the survival and proliferation of activated CD4^+^ T cells. IL-2 levels are disturbed during HIV-1 infection, but the underlying mechanism(s) requires further investigation. We have reported that cellular microRNA (miRNA) let-7i upregulates IL-2 expression by targeting the promoter TATA-box region, which functions as a positive regulator. In this study, we found that HIV-1 infection decreases the expression of let-7i in CD4^+^ T cells by attenuating its promoter activity. The reduced let-7i miRNA expression led to a decline in IL-2 levels. A let-7i mimic increased IL-2 expression and subsequently enhanced the resistance of CD4^+^ T cells to HIV-1-induced apoptosis. By contrast, the blockage of let-7i with a specific inhibitor resulted in elevated CD4^+^ T cell apoptosis during HIV-1 infection. Furthermore, by knocking down the expression of IL-2, we found that the let-7i-mediated CD4^+^ T cell resistance to apoptosis during HIV-1 infection was dependent on IL-2 signaling rather than an alternative CD95-mediated cell-death pathway. Taken together, our findings reveal a novel pathway for HIV-1-induced dysregulation of IL-2 cytokines and depletion of CD4^+^ T-lymphocytes.

The causes of CD4^+^ T cell depletion in acquired immunodeficiency syndrome (AIDS) patients have not been fully elucidated. Several predisposing factors have been reported to contribute to HIV-1-induced CD4^+^ T cell death^1^. For example, viral proteins, including Tat, Nef, Vpr and Env, can induce cell death^2^,^3^,^4^,^5^. The integration of proviral DNA into the chromosome is also a trigger of cell death^6^. Recently, Doitsh *et al*. reported that most CD4^+^ T cell death during HIV-1 infection are caused by caspase-1-mediated pyroptosis triggered by abortive viral infection^7^. They further demonstrated that the incomplete viral transcripts are sensed by interferon-γ-inducible protein 16 (IFI16) and trigger the activation of caspase-1 and pyroptosis^8^.

Interleukin-2 (IL-2) is a key cytokine that regulates the proliferation, differentiation and survival of T cells^9^. By promoting the differentiation of T cells into effector T cells, memory T cells and T helper cells following stimulation with an antigen, IL-2 activates immune responses to help the host counteract the invasion of pathogens^10^. IL-2 is mainly secreted by activated CD4^+^ T cells, and its expression is regulated by a complex network involving transcription factors, chromatin remodeling and CD28 costimulation signaling^11^. It has been reported that HIV-1 infection of CD4^+^ T cells leads to abnormal expression of the IL-2 gene and disturbs the efficient anti-viral immune responses mediated by IL-2^12^,^13^,^14^,^15^,^16^. The gradual loss of IL-2 secretion and proliferation is an early sign of T cell exhaustion in HIV-1 infection^17^. IL-2 is also key for maintaining the viability of activated CD4^+^ T cells by inducing *bcl-2*, *c-myc* and other genes^18^,^19^. The administration of IL-2 to HIV-1-infected individuals could significantly increase CD4^+^ T cell counts compared with antiretroviral therapy alone^20^,^21^,^22^. However, the mechanism of dysregulation of IL-2 during HIV-1 infection and its correlation with the depletion of CD4^+^ T cells have not been properly investigated^23^,^24^.

MicroRNAs represent an important regulator of gene expression in metazoans^25^,^26^. Most miRNAs downregulate gene expression by suppressing translation or inducing degradation of mRNA via targeting the 3′ UTR^27^,^28^,^29^. In recent years, it has been shown that miRNAs can also activate gene transcription through targeting gene promoter regions^30^,^31^. In addition, we revealed that a novel HIV-1-encoded miRNA, miR-H3, could specifically target the TATA-box motif in the HIV-1 5′ LTR and enhance viral gene transcription and viral replication^32^. To address the question of whether this is a virus-specific gene regulatory mechanism, our recent work demonstrated that cellular miRNA let-7i could also activate IL-2 gene transcription through targeting the promoter TATA-box region and functions as a positive regulator of IL-2 gene expression^33^. In addition, the impaired expression of several let-7 family members has been observed in chronic HIV-1-infected patients^34^. Accordingly, we hypothesized that HIV-1 infection could reduce the IL-2 expression by downregulating let-7i miRNA, leading to the death of both infected and bystander activated CD4^+^ T cells.

## Results

### HIV-1 infection decreases IL-2 production in CD4^+^ T cells

**Figure 1:**
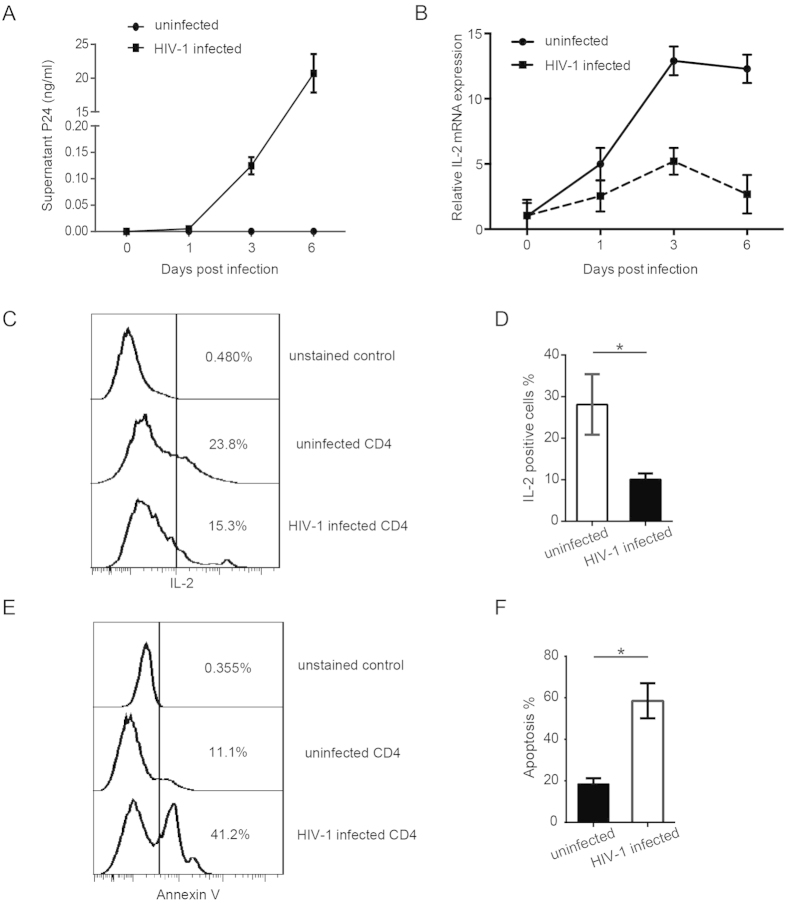
IL-2 expression significantly decreased in HIV-1-infected CD4^+^ T cells. Activated primary blood CD4^+^ T cells (1 × 10^6^) were infected with HIV-1_NL4-3_ viruses at equivalent to 5 ng of p24. (**A**) P24 levels in the supernatants of HIV-1_NL4-3_ infected or uninfected CD4^+^ T cell cultures were examined by ELISA at multiple time points post-infection as indicated. (**B**) *IL-2* mRNA levels in HIV-1-infected or -uninfected CD4^+^ T cells were measured by real-time quantitative RT-PCR at multiple time points post-infection as indicated. A combination of GAPDH, β-actin, RPL13A and IPO8 reference gene mRNA was used as internal control. The *IL-2* mRNA level at each time point was normalized to the uninfected sample of D0. These data represent three independent experiments. (**C**) Intracellular IL-2 protein levels in HIV-1-infected or -uninfected CD4^+^ T cells at day 3-post infection were analyzed by flow cytometry (FCM). The IL-2 positive cells were gated by unstained cell control. (**D**) Statistic analysis of (**C**) was done with data from 6 independent experiments. Paired, two-tailed student’s t test: *p < 0.05. (**E**) Apoptosis of HIV-1_NL4-3_ infected or uninfected CD4^+^ T cells was measured at day 3-post infection using Annexin-V staining method. The Annexin V positive cells were gated by unstained cell control. (**F**) Statistic analysis of (**E**) was done with data from 6 independent experiments. Paired, two-tailed student’s *t* test: *p < 0.05.

Several previous studies reported defective IL-2 secretion in patients with progressive HIV infection compared with elite controllers or healthy controls^13^,^14^,^15^,^16^, but very few studies have assessed the mechanism(s) of this dysregulation by investigating the change in CD4^+^ T cell IL-2 production following the onset of viral infection *in vitro*. To determine how HIV-1 infection affects the IL-2 level in CD4^+^ T cells, primary human blood CD4^+^ T cells were infected with wild-type infectious HIV-1_NL4-3_ viruses. CD4^+^ T cells were isolated from the peripheral blood mononuclear cells (PBMCs) of healthy donors and then activated with anti-CD3 and anti-CD28 before infection. The successful infection of CD4^+^ T cells was confirmed by detecting p24 antigen in the supernatant at several time points post infection (Fig. 1A). The data showed that, along with the infection, the IL-2 mRNA level of CD4^+^ T cells was gradually reduced compared to the uninfected cells (Fig. 1B). The results were consistent when normalized the IL-2 mRNA levels with housekeeping genes (GAPDH + β-actin) or other reference genes (IPO8 + RPL13A, Supplementary Fig. S1). IL-2-secreting CD4^+^ T cells detected by flow cytometry (FCM) were also significantly reduced during HIV-1 infection (Fig. 1C,D). Because IL-2 is a very important cytokine for maintaining activated CD4^+^ T cell survival and proliferation, we examined the depletion of CD4^+^ T cells during HIV-1 infection by both cell morphology and Annexin V staining assay. The dead CD4^+^ T cells have a very distinct morphology (lower FSC, higher SSC) compared to live cells (higher FSC, lower SSC)^35^. And the Annexin V staining can detect early stages of apoptosis (the dying cells)^36^. We observed the expected elevation of CD4^+^ T cell apoptosis concomitant with HIV-1 infection (Fig. 1E,F; Supplementary Fig. S2), which is consistent with the decreased IL-2 level.

### Let-7i activates IL-2 expression in human CD4^+^ T cells

**Figure 2:**
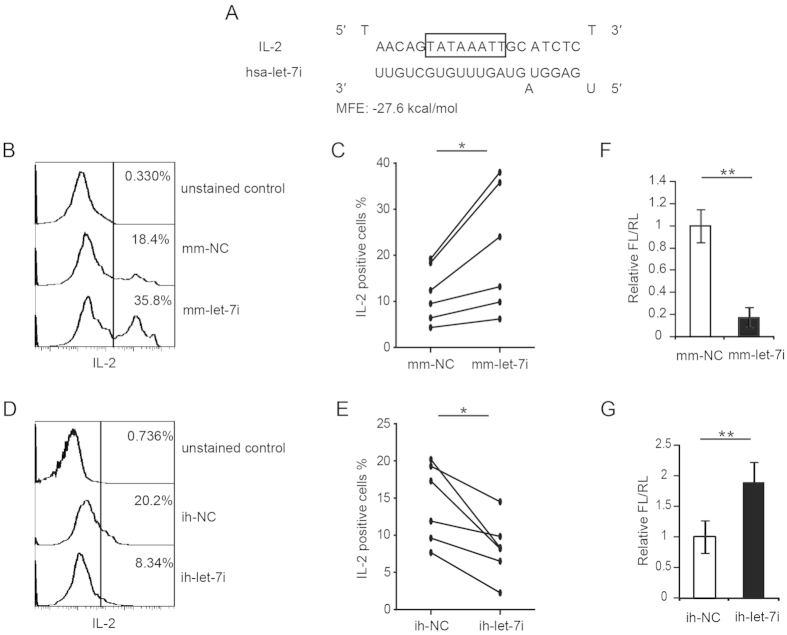
Let-7i upregulates IL-2 expression by targeting the TATA-box region of promoter. (**A**) Predicted binding between let-7i and the TATA box region of human IL-2 gene promoter. MFE: minimum free energy. (**B**) Intracellular IL-2 levels in HIV-1-infected CD4^+^ T cells transfected with let-7i mimics were measured by FCM. The IL-2 positive cells were gated by unstained cell control. (**C**) Statistical analysis of (**B**) was done with data from 6 independent experiments. Paired, two-tailed student’s *t* test: *p < 0.05. (**D**) Intracellular IL-2 levels in HIV-1-infected CD4^+^ T cells transfected with let-7i inhibitors were measured by FCM. (**E**) Statistical analysis of (**D**) was done with data from 6 independent experiments. Paired, two-tailed student’s *t* test: *p < 0.05. (**F**,**G**) HEK293T cells were transfected with a plasmid containing firefly luciferase with let-7i binding site at the 3′ UTR (pMIR-REPORT-let-7i_bindingsite) and a renilla luciferase-expressing plasmid. Cells were co-transfected with let-7i mimic (**F**) or let-7i inhibitor (**G**). Dual luciferase activity was measured at 48 h after transfection. Statistical analysis was done with data from three independent experiments with triplicate samples. Paired, two-tailed student’s t test: **p < 0.01. Mm-, miRNA mimic; ih-, miRNA inhibitor. NC, negative control.

We previously demonstrated that cellular miRNA let-7i could activate the expression of the IL-2 gene by increasing its promoter transcription activity in uninfected activated CD4^+^ T cells^33^. Here we tried to examine whether let-7i can activate the IL-2 expression in HIV-1-infected CD4^+^ T cells. Figure 2A shows the putative binding between let-7i and the IL-2 promoter TATA-box region. In absence of HIV-1 infection, the overexpression of let-7i by transfecting miRNA mimics significantly increased the ratio of IL-2-producing CD4^+^ T cells (Supplementary Fig. S3A). Similarly, in presence of HIV-1 infection, the let-7i miRNA mimic transfection also significantly increased the ratio of IL-2-producing CD4^+^ T cells (Fig. 2B,C). By contrast, when endogenous let-7i was blocked by a specific antisense inhibitor, the fraction of IL-2 positive cells was reduced for both uninfected (Supplementary Fig. S3B) and HIV-1-infected CD4^+^ T cells (Fig. 2D,E). These results indicate that cellular let-7i is a positive regulator of IL-2 production in CD4^+^ T cells. As controls, the efficiency of both the let-7i mimic and inhibitor was confirmed by a luciferase reporter assay carried out in HEK293T cells. These data showed that the let-7i mimic or inhibitor could significantly suppress or enhance the luciferase activity with a perfect let-7i binding site in its 3′ UTR (Fig. 2F,G).

### HIV-1 infection suppresses let-7i expression by attenuating its transcription

**Figure 3:**
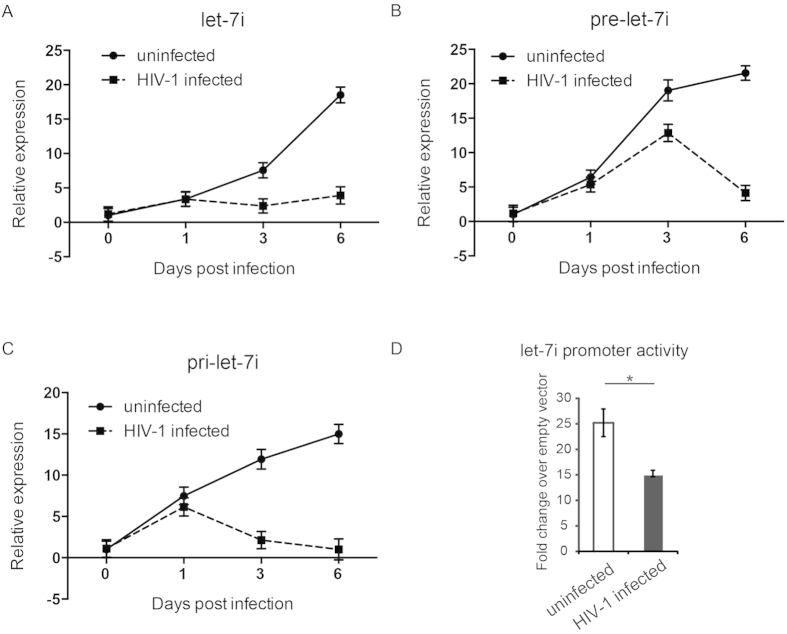
HIV-1 infection significantly reduces primary, precursor and mature let-7i expression in CD4^+^ T cells. (**A**) Mature let-7i levels in HIV-1_NL4-3_ infected or uninfected CD4^+^ T cells were measured by stem-loop real-time RT-PCR. A combination of GAPDH, β-actin, RPL13A and IPO8 mRNA was use as internal control. The let-7i level at each time point was normalized to that of D0 uninfected sample. (**B**) Primary let-7i levels during HIV-1 infection were analyzed by real-time RT-PCR, and normalized by the same way as (**A**). (**C**) Precursor let-7i levels measured during HIV-1 infection by real-time RT-PCR, and normalized by the same way as (**A**). (**D**) HeLa-CD4 cells were transfected with let-7i promoter reporter (pGL4-let-7i) and a renilla luciferase-expressing plasmid at 48 hrs post HIV-1_NL4-3_ infection. After another 48 hrs, the let-7i promoter activity was detected by dual luciferase assay. Statistical analysis was done with data from three independent experiments. Paired, two-tailed student’s *t* test: *p < 0.05. All these data represent at least three independent experiments with triplicate samples.

We then examined whether HIV-1 infection affects the level of let-7i in CD4^+^ T cells. We infected primary human CD4^+^ T cells with HIV-1_NL4-3_ virus as described above and found that, along with the infection, the levels of mature let-7i miRNA in CD4^+^ T cells decreased gradually, which is in agreement with the IL-2 expression change during HIV-1 infection (Fig. 3A). To further explore the mechanism(s) of how HIV-1 infection affects let-7i expression, we also tested the levels of primary and precursor let-7i transcripts in HIV-1-infected CD4^+^ T cells. Our results showed that the expressions of both primary and precursor let-7i were also repressed by HIV-1 infection (Fig. 3B,C). These data suggest that HIV-1 infection could inhibit the transcription of *let-7i*.

To test this hypothesis, we used a promoter activity reporter system, pGL4-let-7i, containing a full-length let-7i promoter (~2500 bp)-driven luciferase gene^37^, which was provided by Dr. Ashish Lal at the National Cancer Institute, USA^38^. The HeLa-CD4 cells were initially infected by HIV-1_NL4-3_, and the cells were then transfected with pGL4-let-7i and the *Renilla* luciferase control reporter vector pRL-TK at two days post infection. The dual-luciferase reporter assay data indicated that, compared to uninfected controls, HIV-1 infection indeed repressed the let-7i promoter activity (Fig. 3D).

### Let-7i reduces CD4^+^ T cells apoptosis induced by HIV-1 infection

**Figure 4:**
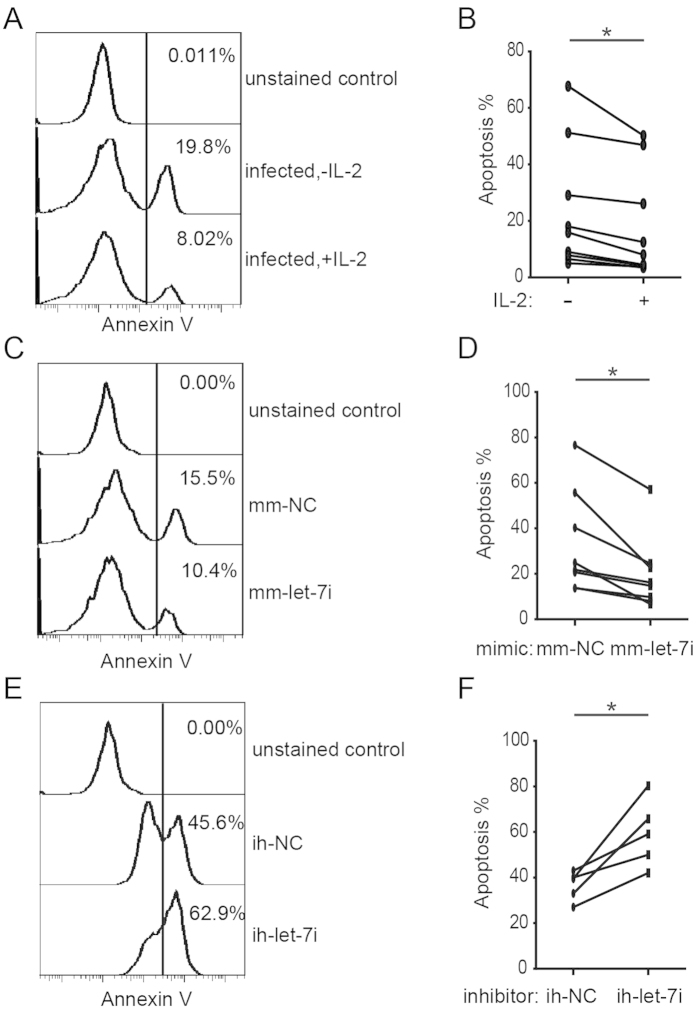
Let-7i increases resistance to apoptosis in HIV-1-infected CD4^+^ T cells. Activated human CD4^+^ T cells were treated with IL-2 or transfected with let-7i mimic/control or let-7i inhibitor/control oligonucleotides. Cells were then infected with HIV-1_NL4-3_ as described above. (**A**) Annexin V staining for apoptosis of IL-2 treated CD4^+^ T cells during HIV-1_NL4-3_ infection were analyzed by FCM 24-48 hrs post infection. (**B**) Statistical analysis of (**A**) was done with data from 9 independent experiments. (**C**) Annexin V staining for apoptosis of HIV-1-infected CD4^+^ T cells transfected with let-7i mimic or negative control (mm-NC) were measured by FCM 24-48 hrs post infection. (**D**) Statistical analysis of (**C**) was done with data from 8 independent experiments. (**E**) Annexin V staining for apoptosis of HIV-1-infected CD4^+^ T cells treated with let-7i inhibitor or negative control (ih-NC) were analyzed by FCM 24-48 hrs post infection. (**F**) Statistical analysis of (**E**) was done with data from 5 independent experiments. All the statistical analysis was done with paired, two-tailed student’s *t* test: *p < 0.05.

Collectively, our results have shown that HIV-1 infection could induce the suppression of both IL-2 and let-7i expression. Given that let-7i is a positive regulator of IL-2 gene transcription, it is possible that suppression of let-7i contributes to the CD4^+^ T cell death caused by HIV-1 infection. To address this question, let-7i was overexpressed or blocked in CD4^+^ T cells, and the cells were then infected with HIV-1_NL4-3_ viruses. We first confirmed the effects of IL-2 in maintaining CD4^+^ T cell survival in HIV-1 infection. The data showed that IL-2 could reduce the apoptosis caused by viral infection as shown by both Annexin V staining and morphological data (Fig. 4A,B; Supplementary Fig. S4A), which is consistent with previous studies^20^,^22^,^39^,^40^. We then tested the effects of let-7i on CD4^+^ T cell survival; similarly, the overexpression of let-7i also reduced the apoptosis of infected CD4^+^ T cells (Fig. 4C,D; Supplementary Fig. S4B). By contrast, when let-7i was inhibited by a specific inhibitor, the apoptosis level of infected CD4^+^ T cells increased (Fig. 4E,F; Supplementary Fig. S4C).

As controls, similar experiments were carried out with uninfected CD4^+^ T cells (Supplementary Fig. S5). In absence of HIV-1 infection, overexpression of let-7i still protected CD4^+^ T cells against apoptosis (Supplementary Fig. S5D–F), which might be triggered by activation-induced cell death (AICD). This observation resembled the treatment of IL-2 (Supplementary Fig. S5A–C). And the treatment with the let-7i miRNA inhibitor showed opposite effects (Supplementary Fig. S5G–I), although only a small effect on increasing T cell death of uninfected cells was observed. Taken together, the let-7i induced IL-2 has a protective effect against CD4^+^ T cell apoptosis, especially protects cells from the cytotoxicity caused by HIV-1 infection.

### Let-7i-mediated resistance to apoptosis is dependent on the IL-2 signaling pathway

**Figure 5:**
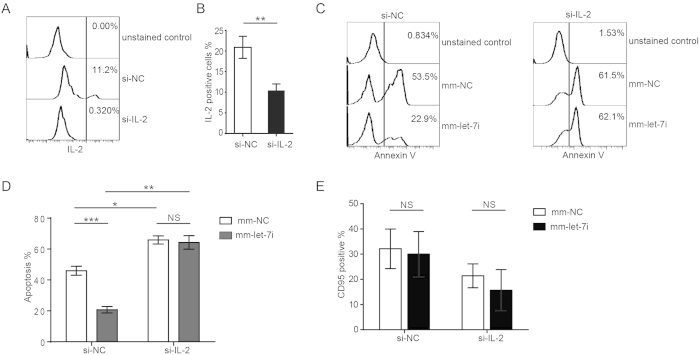
The resistance to apoptosis in HIV-1 infected CD4^+^ T cells endowed by let-7i is dependent on IL-2 expression. (**A**) Silencing of IL-2 by IL-2-specific siRNAs was confirmed by FCM analysis on IL-2-producing CD4^+^ T cells. (**B**) Statistical analysis of (**A**) was done with data from three independent experiments. Paired, two-tailed student’s t test, **p < 0.01. (**C**) Human CD4^+^ T cells were transfected with let-7i mimic, siRNA against IL-2 or respective controls (mm-NC, si-NC). Cells were then infected with HIV-1_NL4-3_ as described above. Apoptosis of CD4^+^ T cells were measured by Annexin V staining. (**D**) Statistical analysis of (**C**) was done with data from three independent experiments. Paired, two-tailed student’s t test: *p < 0.05; **p < 0.01, ***p < 0.001. (**E**) Human CD4^+^ T cells were treated as in (**C**), and CD95 expression was measured by FCM. Statistical analysis was done with data from three independent experiments. Paired, two-tailed student’s t test. NS, non-significant.

To further confirm that let-7i-mediated CD4^+^ T cell resistance to HIV-1-induced cell death is via the IL-2 signaling pathway, we used siRNAs specifically targeting IL-2 to silence its expression. The FCM data showed that the expression of IL-2 was successfully reduced by its specific siRNA, siIL-2 (Fig. 5A,B). We then examined the effects of let-7i on HIV-1-induced CD4^+^ T cell apoptosis following IL-2 knockdown. In the control group, the let-7i mimic still significantly decreased the HIV-1 infection-induced apoptosis of CD4^+^ T cells (Fig. 5C,D, left panel), but when IL-2 was knocked down, the apoptosis caused by HIV-1 infection was not rescued by let-7i overexpression (Fig. 5C,D, right panel). Compared with the negative control, the knockdown of IL-2 substantially increased the apoptosis levels of infected CD4^+^ T cells (Fig. 5D).

It has been suggested that the death receptor Fas (CD95) is a target of let-7 family miRNAs^41^,^42^. To exclude the possibility that let-7i protects HIV-1-infected CD4^+^ T cells from apoptosis by downregulating CD95 expression, we also examined CD95 expression when let-7i was overexpressed. In either IL-2 knockdown or control CD4^+^ T cell groups, CD95 expression was not significantly affected by the overexpression of let-7i (Fig. 5E). Therefore, let-7i-mediated resistance to HIV-1-induced apoptosis is independent of the CD95-mediated apoptosis pathway^43^.

## Discussion

IL-2 is a crucial cytokine secreted by activated T lymphocytes and a major regulator of anti-apoptotic function. Our data show that HIV-1 infection could decrease the IL-2 expression of CD4^+^ T cells *in vitro* (Fig. 1). This finding is consistent with several other studies indicating that the HIV-1 infection reduces IL-2 levels in HIV-1-infected patients^13^,^14^,^15^,^16^. Seddiki *et al*. reported that a reduction in IL-2 expression in chronically HIV-infected (CHI) patients was accompanied by generalized T cell dysfunction^13^. In another study, Fan *et al*. also found lower IL-2 mRNA levels in HIV-seropositive subjects^14^. Interestingly, in a follow-up study, they found that men whose CD4^+^ T cell levels fell more than 200/mm^3^ in the following 2 years showed lower IL-2 levels compared to those with unchanged CD4^+^ T cell levels^14^, implying a correlation between the decrease in IL-2 levels and the drop in CD4^+^ T cell counts. Many host factors are involved in the regulation of IL-2 expression, such as transcription modulator BCL11B, Ikaros and the CD28 costimulation-signaling pathway^44^,^45^,^46^,^47^. However, very few studies have investigated the mechanism of how HIV-1 infection alters the expression of IL-2^13^. Our recent work indicated that miRNA let-7i binds to the TATA-box region of the IL-2 promoter and activates gene transcription in CD4^+^ T cells by facilitating the assembly of pre-initiation complexes for mRNA transcription^33^. These findings suggest that let-7i is an activator of IL-2 expression, but the physiological significance of this pathway is still elusive. We addressed this question in the present study.

HIV-1 infection has been shown to interfere with the RNA silencing pathway, including alteration of the expression of many host miRNAs^48^,^49^,^50^. In this study, our data show that HIV-1 infection led to the reduction of mature let-7i levels, as well as reduction of the precursor and the primary let-7i levels (Fig. 3A–C). Further experiments indicated that the promoter activity of let-7i was significantly decreased in HIV-1-infected cells (Fig. 3D). It has been reported that HIV-1 infection induces downregulation of let-7 miRNAs in a T cell model and in chronic HIV-1-infected patients^34^, but the mechanism of how HIV-1 infection affects let-7 miRNA expression has not been fully elucidated. During the infection of human biliary epithelial cells (cholangiocytes) by the protozoan parasite *Cryptosporidium parvum*, the let-7i promoter activity was decreased^37^. O’Hara *et al*. showed that microbial infection promotes the formation of a NFκB p50-C/EBPβ silencer complex on the regulatory sequences of the let-7i promoter and promotes histone-H3 deacetylation^37^. In human cancer cells, mutant p53 downregulates the transcription of let-7i through p63^38^. It was therefore rational to investigate whether the HIV-1 infection-induced reduction of let-7i promoter activity is achieved through similar pathways. Additionally, it is of interest to investigate whether specific multifunctional viral proteins, such as Tat and Nef, are involved in this process.

CD95 (Fas) triggers apoptosis in a variety of cells^51^. CD95 has been reported to be a target of let-7 family miRNAs, and the introduction of let-7 could decrease the sensitivity to Fas-induced apoptosis^41^. However, in our study, the treatment of CD4^+^ T cells with let-7i did not significantly decrease the level of cell-surface CD95 (Fig. 5E). The silencing of IL-2 by siRNA diminished the let-7i-mediated resistance of CD4^+^ T cells to HIV-1 infection-induced apoptosis (Fig. 5A–D). These data suggest that the let-7i-induced CD4^+^ T cell survival is mainly through the upregulation of IL-2 rather than the downregulation of CD95 in HIV-1-infected CD4^+^ T cells. In summary, our study indicates that HIV-1 infection leads to the downregulation of the let-7i/IL-2 axis and contributes to CD4^+^ T cell death. These results reveal a novel pathway for HIV-1-induced CD4^+^ T cell death, which exploits the function of IL-2 as a key cytokine to maintain the viability of activated T cells. In addition, because IL-2 is also a crucial cytokine that controls the balance of immune responses, the let-7i/IL-2 axis might also be involved in other immune dysregulation observed during HIV-1 infection, such as anergy and exhaustion of T cells^17^,^52^,^53^,^54^.

Many factors contribute to the depletion of CD4^+^ T cells in HIV-1 infection, including viral cytotoxic factors, host anti-infection immune response and aberrant immune activation, which adds to normal immune defense mechanisms^55^. In this report, we utilized an *in vitro* peripheral blood CD4 T cell model to investigate the cell death pathway related to HIV-1-induced IL-2 dysregulation. Because the activated CD4^+^ T cells support productive infection of HIV-1, the triggers of cell death here are most likely direct viral cytotoxicity and/or activation-induced cell death (AICD) as shown in uninfected CD4^+^ T cell experiments (Supplementary Fig. S5). Multiple HIV-1 viral proteins and replication steps, such as Vpr, Tat, Env, reverse transcription and integration, can induce apoptosis of infected CD4^+^ T cells (see review^55^). Recently, Trinité *et al*. reported that Vpr- and reverse transcription-induced apoptosis in resting peripheral blood CD4 T cells can be inhibited by common gamma-chain cytokines (CGCC), such as IL-7 and IL-4^35^, which may account for the resting CD4^+^ T cell resistance to infection. This study shed light on the mechanism of how cytokines are involved in HIV-associated blood T cell death. Likewise, in this study we showed that HIV-1 infection suppresses IL-2 through let-7i, which greatly impaired the protective effect against viral infection- and activation-induced apoptosis of the peripheral blood CD4^+^ T cells by IL-2. Doitsh *et al*. reported the death of lymphoid T cells caused by abortive infection-induced pyroptosis, which is triggered by the cellular sensor IFI16^7^,^8^. However, this pyroptosis mechanism is unlikely to play a major role in the depletion of peripheral blood CD4 T cells because IFI16 is not expressed in peripheral blood T cells^56^.

There have been reports of IL-2 elevation during HIV-1 acute infection, which may be a component of the so called “cytokine storm” during acute infection of HIV^57^. This is a reasonable observation because during acute HIV infection, there is widespread activation and rapid expansion of lymphocytes, including DC, NK, HIV-1-specific CD4^+^ T cells and CD8^+^ T cells, which express high levels of activation markers, such as CD38 and human leukocyte antigen (HLA)-DR^54^. IL-2 signals are important for CD8^+^ T cell proliferation during acute HIV-1 infection^58^. Due to the strong stimulation of the pathogen during acute infection, cytokine levels are expected to increase to coordinate an effective immune response. At the same time, the intense cytokine response during acute HIV infection may also promote viral replication and lead to immunopathology^54^. Thus, the IL-2 elevation during acute infection is not sufficient to block the massive depletion of CD4 T cells caused by the strong cytotoxic effects of the innate immune response and the substantial cytotoxicity due to peak viremia. For chronic progressive HIV-1 infection, several studies have reported reduced IL-2 levels in HIV infected individuals compared with long-term non-progressors or healthy controls^13^,^15^,^34^. These findings indicate that the reported mechanism resembles the situation of chronic progressive HIV-1 infection more than the acute infection stage. During chronic progressive HIV-1 infection, there is also progressive loss of CD4^+^ lymphocytes and perturbation of immune function, which may be due to the dysregulation of IL-2 and other cytokines through unrevealed pathways.

## Methods

### Ethics Statement

This research was approved by the Ethics Review Board of Sun Yat-Sen University. All experiments were performed in accordance with relevant guidelines and regulations. Written informed consent was provided by all study participants and/or their legal guardians.

### Cell culture

Peripheral blood mononuclear cells (PBMCs) from HIV-seronegative donors were isolated by Ficoll gradient centrifugation (TBD Science, Tianjin, China). The CD4^+^ T cells were then separated with the Human CD4^+^ T Lymphocyte Enrichment Set-DM (BD IMag™) according to the manufacturer’s instructions. The selected cell populations contained more than 95% CD3^+^ CD4^+^ T cells as measured by flow cytometry. CD4^+^ T cells were then cultured in RPMI 1640 conditioned medium supplemented with 10% fetal bovine serum, 100 units/ml of penicillin, and 100 μg/ml of streptomycin. Subsequently, the primary CD4^+^ T cells were stimulated with anti-CD3 (1 μg/ml) and anti-CD28 (1 μg/ml) for 48 hrs before HIV-1 infection and/or small RNA transfection experiments. HEK293T cells were obtained from ATCC (American Type Culture Collection, Manassas, VA) and HeLa-CD4 cells were obtained from the AIDS Research and Reference Reagent Program, NIAID, NIH, USA. HEK293T cells and HeLa-CD4 cells were maintained in the conditioned Dulbecco’s modified Eagle’s medium (DMEM) supplemented with 10% fetal bovine serum, 100 units/ml of penicillin, and 100 μg/ml of streptomycin.

### Plasmids, antibodies, miRNA mimics and inhibitors

The infectious HIV-1 clone (pNL4-3) was obtained through the AIDS Research and Reference Reagent Program, NIAID, NIH, USA. The let-7i promoter reporter (pGL4-let-7i) was kindly bestowed by Dr. Ashish Lal (Genetics Branch, National Cancer Institute, National Institutes of Health, Bethesda, MD, USA). pRL-TK renilla luciferase control reporter vector was from Promega (USA). A perfect binding sequence of let-7i was inserted into the 3′ UTR of luciferase gene of the pMIR-REPORT™ miRNA Expression Reporter Vector (Promega, USA) to generate pMIR-REPORT-let-7i_bindingsite construct.

Anti-human CD3 and anti-human CD28 antibodies were from BD Biosciences (Palo Alto, CA). The anti-IL-2 and anti-CD95 antibodies for flow cytometry analysis were from BD Biosciences (Palo Alto, CA). Annexin-V cell apoptosis assay kit was from Keygen (Nanjing, China).

The let-7i mimics and negative control (mm-NC) were purchased from RiboBio (Guangzhou, China). let-7i mimic sequence: 5′-UGAGGUAGUAGUUUGUGCUGUU-3′; NC mimic sequence: 5′-UUUGUACUACACAAAAGUACUG-3′. The let-7i antisense inhibitor and control were purchased from GenePharma (Shanghai, China). Small interfering RNAs (siRNAs) smart pool used to specifically target human *IL-2* mRNA were synthesized by RiboBio (Guangzhou, China).

### Flow cytometry

For flow cytometry analysis of IL-2, CD4^+^ T cells were stimulated with anti-CD3 (1 μg/ml) and anti-CD28 (1 μg/ml) for 48 hrs before small RNA transfection. The HIV-1 infection was carried out at 6 hrs post transfection. After 48 hrs of small RNA transfection, CD4^+^ T cells were labeled with anti-human IL-2-PE-conjugated mAbs after fixation and permeabilization using the BD Cytofix/Cytoperm™ Fixation/Permeabilization Kit (Cat. No. 554714) and BD GolgiPlug™ protein transport inhibitor (Cat. No. 555028) by following the manufacturer’s instructions. For the experiments that the CD4^+^ T cells were only infected with HIV-1 without small RNA treatments, the CD4^+^ T cells were collected at day 3 post infection for IL-2 expression assay with FCM. Alternatively, cells were stained and washed followed by immediate flow cytometry analysis (FCM) for CD95 staining. For Annexin V staining assay of cell apoptosis, CD4^+^ T cells were stimulated with anti-CD3 (1 μg/ml) and anti-CD28 (1 μg/ml) for 48 hrs before small RNA transfection, then the cells were infected with HIV-1 viruses 6 hrs later post transfection. The infected cells were collected 24–48 hrs post HIV-1 infection for Annexin V staining to observe the peak effect of small RNAs. For the experiments that the CD4^+^ T cells were only infected with HIV-1 without small RNA treatments, the infected and uninfected control cells were collected at 72 hrs post infection. A PE-conjugated Annexin-V apoptosis kit was used for cell apoptosis detection according to the manufacturer’s instructions. Briefly, morphologically dead cells were gated out and only live cells were used for Annexin V staining assay. For gating the Annexin V positive cells, unstained live cells were used as negative control. Mean fluorescent intensities (MFI) were measured by FCM (LSRFsortessa, BD) and data were analyzed by using FlowJo software (Tree Star).

### Real-Time Quantitative PCR

Total RNA from CD4^+^ T cells was isolated using TRIzol reagent (Invitrogen), followed by DNase digestion (TURBO DNA-free, Ambion) and then subjected to cDNA synthesis using PrimeScript RT reagent Kit (Takara). Quantitative PCR was performed using SYBR Premix ExTaq II Kit (Takara) in CFX96™ Real-Time PCR Detection System (Bio-Rad). Quantification of IL-2 mRNA and let-7i miRNAs were normalized to a combination of GAPDH, β-actin, RPL13A and IPO8 reference gene. The primer sets used were as follows: *IL-2*-F, 5′-GAACTAAAGGGATCTGAAACAACATTC-3′, *IL-2*-R, 5′-TGTTGAGATGATGCTTTGACAAAA-3′; let-7i-F, 5′-GCGGCGGTGAGGTAGTAGTTTGT-3′, let-7i-stem-loop RT, 5′-GTCGTATCCAGTGCAGGGTCCGAGGTATTCGCACTGGATACGACAACAGC-3′; primary let-7i transcript: pri-let-7i-F, 5′-TGCCTCCCCGACACCATG-3′, pri-let-7i-R, 5′-GGATTCCCAGCCATTGTCC-3′; let-7i microRNA precursor: pre-let-7i-F, 5′-CTGGCTGAGGTAGTAGTTTG-3′, pre-let-7i-R, 5′-TAGCAAGGCAGTAGCTTG-3′. The relative expression levels were calculated using the comparative C(T) method.

### HIV-1 production and infection

Infectious HIV-1 clone pNL4-3 was amplified with HB101 competent cells (Promega) and transfected into HEK293T cells to produce viruses as described before by our group^32^. Viral production was determined by detecting HIV-1 p24 with enzyme-linked immunosorbent assay (ELISA). The target cells (1 × 10^6^) were infected with the equivalent of 5~10 ng HIV-1 p24 in 1 ml of medium for 3 hrs at 37 °C. Cells were then washed three times with PBS to remove the virus-containing supernatants. Infected cells were maintained in conditioned RPMI 1640 medium. HIV-1 replication was monitored by p24 detection with ELISA.

### Transfection of plasmid, miRNA mimics and inhibitors

Transfection of HEK293T and HeLa-CD4 cells were performed with Lipofectamine 2000 (Invitrogen) according to the manufacturer’s protocol. Transfection of primary CD4^+^ T cells with small RNAs was performed with RNAiMAX (Invitrogen) according to the manufacturer’s protocol.

### Dual-Luciferase reporter assay

HEK293T cells were seeded in 48-well plates (Corning, China) at 2 × 10^4^ cells per well at the day before transfection. Ten ng of pMIR-REPORT-let-7i_bindingsite and 2 ng of renilla luciferase (RL) constructs were co-transfected into HEK293T cells using Lipofectamine 2000 (Invitrogen) by following the manufacturer’s protocol. After 24–48 hrs, FL and RL activities were measured with the Dual-Glo luciferase assay system according to the manufacturer’s instructions (Promega). HeLa-CD4 cells were seeded in 24-well plates at a density of 2 × 10^4^ cells per well and cultured for overnight. The cells were then infected with the equivalent of 5 ng HIV-1 p24 per well. Forty-eight hours post infection, cells were transfected with 10 ng of let-7i promoter driven-firefly luciferase (FL) reporter (pGL4-let-7i) and 2 ng of renilla luciferase (RL) constructs. FL and RL activities were measured at 48 hrs post transfection.

### Statistical analysis

A paired, two-tailed student′s t test was used to determine the significance of data between differently treated sample groups. Data were considered significant at *p* < 0.05.

## Additional Information

**How to cite this article**: Zhang, Y. *et al*. HIV-1 Infection-Induced Suppression of the Let-7i/IL-2 Axis Contributes to CD4^+^ T Cell Death. *Sci. Rep*. **6**, 25341; doi: 10.1038/srep25341 (2016).

## Supplementary Material

Supplementary Information
