# Supplementary material for: HIV-1 Infection-Induced Suppression of the Let-7i/IL-2 Axis Contributes to CD4+ T Cell Death
Source: Sci Rep. 2016 May 5;6:25341. doi: 10.1038/srep25341 (PMC4857132; doi:10.1038/srep25341)
Supplement: Supplementary Information [file srep25341-s1.pdf]

HIV-1 Infection-Induced Suppression of the Let-7i/IL-2 Axis Contributes to CD4+ T Cell Death

Yijun Zhang[a, b,*,¶], Yue Yin[a, b,$,¶], Shaoying Zhang[a, b], Haihua Luo[a, b,#] , Hui Zhang[a, b,#]

[a]Institute of Human Virology, [b]Key Laboratory of Tropical Disease Control of Ministry of Education, Zhongshan School of Medicine, Sun Yat-sen University, Guangzhou, Guangdong, 510080, China.

Running Head: HIV-1 Infection Downregulates Il-2 Through Let-7i

[#] Address correspondence to Hui Zhang, zhangh92@mail.sysu.edu.cn; or Haihua Luo, luohh@mail.sysu.edu.cn.

Present address: [*]Section of Infectious Diseases, Department of Internal Medicine, Yale University School of Medicine, New Haven, CT, 06520, USA; [$]Department of Basic Research, Shaanxi Provincial Tumor Hospital, Xi'an, Shaanxi, 710000, China;

[¶] Y.Z. and Y.Y. contributed equally to this work.

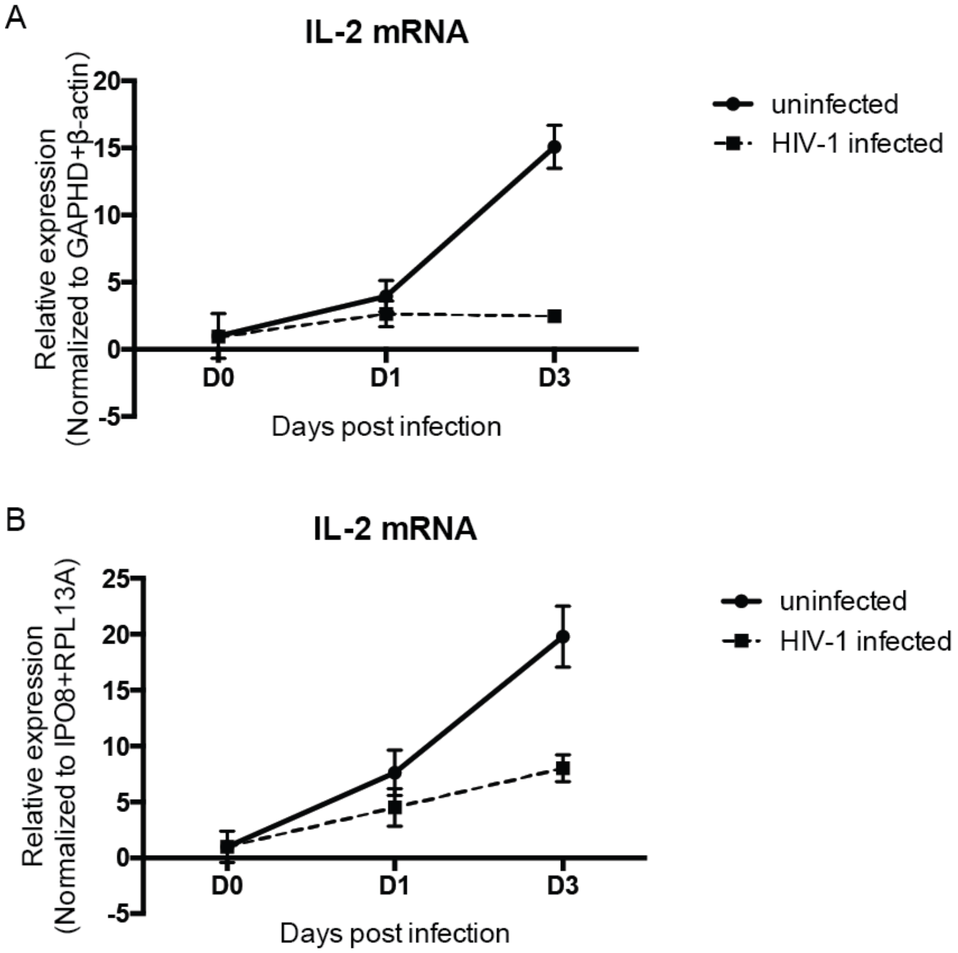

Supplementary Figure S1. IL-2 mRNA levels during HIV-1 infection.

IL-2 mRNA levels in HIV-1-infected or -uninfected $CD4^+$ T cells were measured by real-time quantitative RT-PCR and normalized to GAPDH+ β -actin (A) or IPO8+RPL13A (B) mRNAs at multiple time points post-infection as indicated. These data represent three independent experiments with triplicate samples.

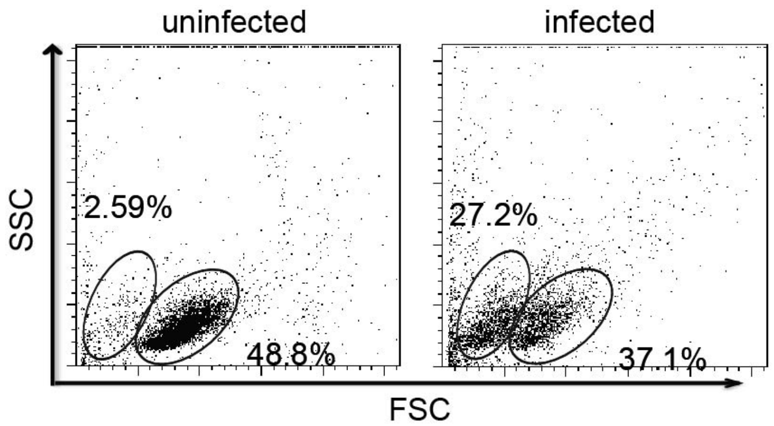

Supplementary Figure S2. Morphological changes of HIV-1 infected CD4$^+$ T CELLS

Forward versus side scatter dot plot (FSC to SSC) of HIV-1-infected and -uninfected CD4$^+$ T cells at day 3 post infection. Live cells (higher FSC, lower SSC) and dead cells (lower FSC, higher SSC) were gated. These data represent at least three independent experiments.

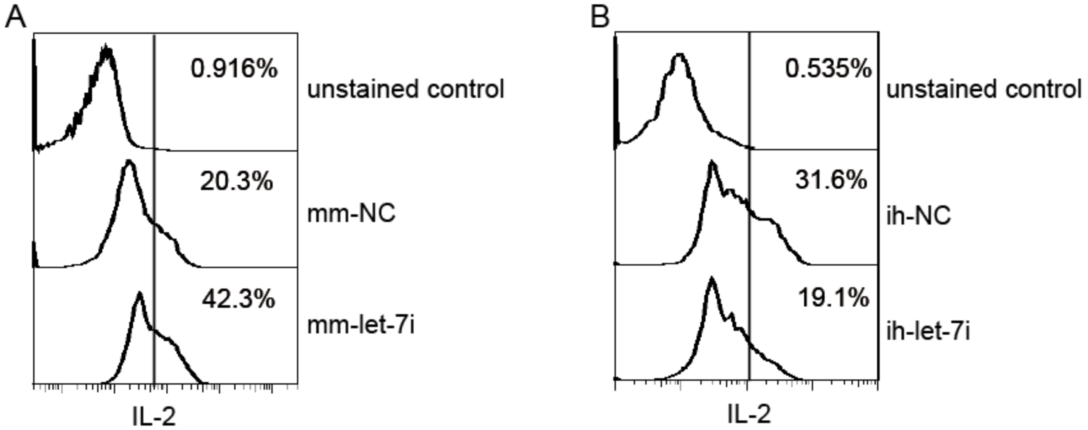

Supplementary Figure S3. Let-7i enhances IL-2 expression in uninfected CD4 T cells.

(A) Intracellular IL-2 levels in uninfected $CD4^+$ T cells transfected with let-7i mimics were measured by FCM. (B) Intracellular IL-2 levels in uninfected $CD4^+$ T cells transfected with let-7i inhibitors were measured by FCM. mm-, miRNA mimic; ih-, miRNA inhibitor. NC, negative control. These data represent at least three independent experiments.

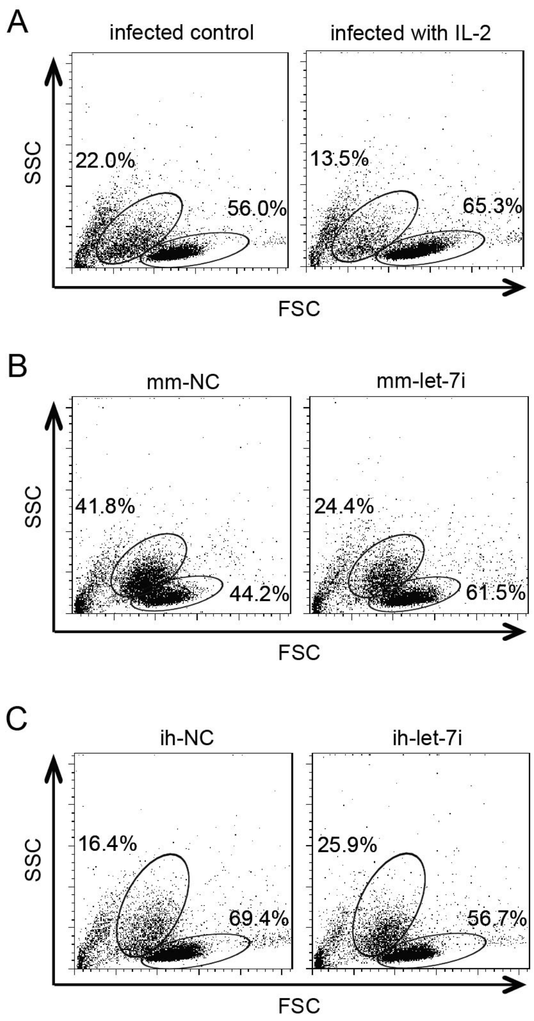

Supplementary Figure S4. Morphological assay of HIV-1-infected CD4⁺ T cells treated with IL-2, let-7i miRNA mimic or inhibitor

(A) Forward versus side scatter dot plot (FSC to SSC) of IL-2 treated $CD4^+$ T cells during $HIV\text{-}1_{NL4\text{-}3}$ infection were analyzed by FCM 24-48 hrs post infection. (B) FSC vs. SSC plot of HIV-1 infected $CD4^+$ T cells transfected with let-7i mimic or negative control (mm-NC) were measured by FCM 24-48 hrs post infection. (C) FSC vs. SSC plot of HIV-1 infected $CD4^+$ T cells treated with let-7i inhibitor or negative control (ih-NC) were analyzed by FCM 24-48 hrs post infection. All these data represent at least three independent experiments.

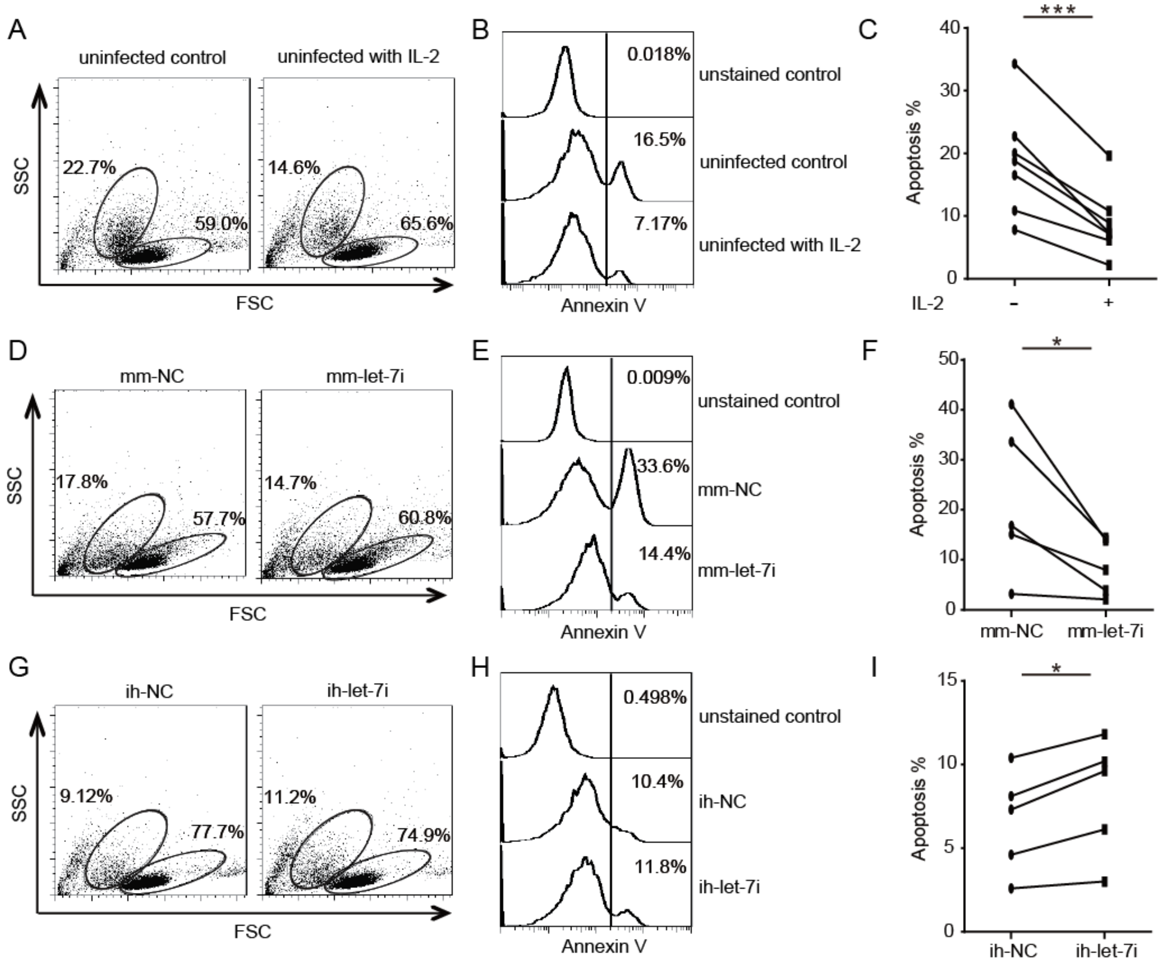

Supplementary Figure S5. Anti-apoptosis effect of let-7i on uninfected CD4^{+} T cells. (A) Forward versus side scatter dot plot (FSC to SSC), (B) Annexin-V staining and (C) statistical analysis (n=7) of Annexin-V staining results of uninfected CD4 T cells in the presence or absence of IL-2 for 48 hrs. (D) Forward versus side scatter dot plot (FSC to SSC), (E) Annexin-V staining and (F) statistical analysis (n=5) of Annexin-V staining results of uninfected CD4 T cells treated with let-7i miRNA mimic or control for 48 hrs. (G) Forward versus side scatter dot plot (FSC to SSC), (H) Annexin-V staining and (I) statistical analysis (n=5) of Annexin-V staining results of uninfected CD4 T cells treated with let-7i miRNA inhibitor or control for 48 hrs. These data represent at least three

independent experiments. Statistical analysis was done with data from at least 5 independent experiments. Paired, two-tailed student's *t* test: *, $p < 0.05$.
